# Green hydrogen futures in LMICs: Opportunities for fertilizer and steel production in Kenya

**DOI:** 10.1016/j.isci.2025.112298

**Published:** 2025-03-27

**Authors:** Pietro Lubello, Joshua Oduor, Anne Nganga, Martin Mutembei, Francis Njoka, Michelle Akute, Kihara Mungai, Steve Pye

**Affiliations:** 1 UCL Energy Institute, University College London, London WC1H 0NN, UK; 2 Deutsche Gesellschaft für Internationale Zusammenarbeit GmbH (GIZ), Nairobi 41607-00100, Kenya; 3 Strathmore Energy Research Center (SERC), Strathmore University, Nairobi 59857-00200, Kenya; 4 Department of Energy, Gas and Petroleum Engineering, Kenyatta University, Nairobi 43844-00100, Kenya; 5 Energy and Petroleum Regulatory Authority (EPRA), Nairobi 42681-00100, Kenya

**Keywords:** Energy resources, Energy policy, Energy engineering

## Abstract

Green hydrogen is often presented as a promising driver of green industrial development in low- and lower-middle-income countries (LMICs), with national strategies balancing local applications promoting sustainable development alongside export opportunities. Country-level energy system models can help identify no-regret options by comparing the costs of local green alternatives to market prices, reducing the risk of uneconomic investments that could hinder local development. We present an open-source capacity expansion model for Kenya to explore the role of green hydrogen in local fertilizer and steel industries under various market and technology development scenarios. With abundant renewable energy resources, we estimate that Kenya could produce hydrogen at 2.7–3.7 USD/kg, making local options competitive from the second half of the 2030s, provided high market prices and substantial additional investments in the development of the sectors. The Kenya case study offers valuable insights for other LMICs as they design and implement their national strategies.

## Highlights

• Analysis of potential future local applications for green hydrogen in Kenya
• Estimated LCOH of 2.7–3.7 USD/kg with optimal power mix varying by location
• GH2 for fertilizer and steel may be competitive from the late 2030s
• Successful adoption relies on developing local industries and supply chains

## Graphical abstract

**Figure:**
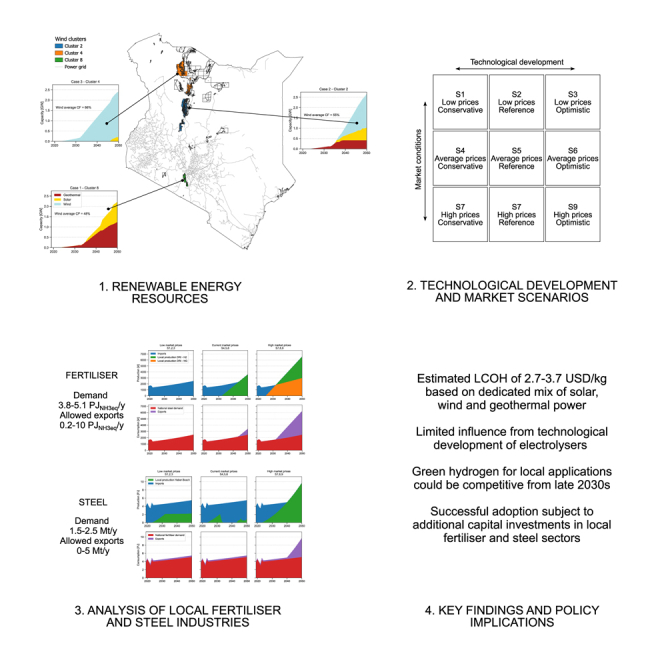

## Introduction

With the emergence of green hydrogen as a key energy vector for the global energy transition, it is increasingly seen as an opportunity for clean development in low- and lower-middle-income countries (LMICs). The potential in Africa is frequently highlighted due to the continent’s enormous renewable energy resources potential and vast land areas.^1^^,^^2^ However, concerns have been raised around risks and barriers to its diffusion. Both Dagnachew et al.^3^ and Tunn et al.^4^ emphasize that the ability to fully harness the potential of green hydrogen for local development is hindered by existing extractivist patterns and current geopolitical realities, reflected in social and ecological risks related to energy, water, land, and social dynamics. Equitable partnerships and investments in technology transfer are proposed as potential strategies to mitigate these risks.^3^ However, other perspectives argue that only broader, more structural changes in the global economic system can provide a viable solution.^4^ From a techno-economic perspective, the successful implementation of national green hydrogen strategies—in LMICs as anywhere else—is highly dependent on cost reductions of renewable energy sources and electrolyzers and when, or whether, green hydrogen will become cost competitive with hydrogen produced from fossil sources or with other decarbonization options in different sectors.^5^^,^^6^ This is particularly relevant considering the emerging implementation gaps in green hydrogen projects globally.^7^

In this context, minimizing risks and ensuring procedural justice in the formulation of national green hydrogen strategies emerge as crucial factors.^8^^,^^9^ A recent report by UNIDO, IRENA, and IDOS emphasizes risk reduction as a central theme,^9^ outlining four key strategic considerations for the effective implementation of green hydrogen strategies aimed at sustainable industrial development: (1) prioritizing local uses before exports, (2) aligning with just transition principles and existing national goals, (3) starting with small- to medium-sized projects, and (4) implementing green hydrogen production and application sequentially. Several national strategies are focused on the identification of “no-regret” options^9^ that lie at the intersection of promising green hydrogen applications and, in line with points (1) and (2), local uses before exports and alignment with existing national goals. In this regard, agriculture and steel production are key underpinning sectors for LMICs that have increasing populations, expanding infrastructure and growing industrial needs. Nitrogen-based fertilizers and steel are generally considered as two of the most promising applications for green hydrogen, both for the level of technological readiness of the processes involved and given the absence of decarbonized alternatives.^5^^,^^6^ Globally, food production heavily depends on nitrogen-based fertilizers and fossil fuel trade. An estimated 1.78 billion people annually rely on imported fertilizers or natural gas (NG), underscoring the sector’s vulnerability to potential supply and energy disruptions.^10^

While green hydrogen is seen as a promising alternative aligned with clean development, it requires more land, energy, and water than conventional production, potentially intensifying land and water scarcity and straining limited natural resources.^10^^,^^11^^,^^12^^,^^13^ For nitrogen-based fertilizers, adopting green hydrogen primarily involves replacing traditional fossil fuel-derived hydrogen with clean hydrogen from electrolysis. In the steel sector, however, successful adoption additionally requires substantial technological advancements in steel production processes. Early deployment of these technologies in leading regions could create spillover effects in later-adopting areas, accelerating widespread implementation.^14^ Depending on coking coal prices required in fossil fuel-based production, green steel could become competitive by 2030 in favorable regions—characterized by high potential of variable renewable energy (VRE) resources, high-quality iron ore, and low steelworker wages—potentially strengthening its position further by 2050.^15^

However, the effective adoption of hydrogen-based fertilizers and green steel in LMICs still depends on these options achieving cost parity with traditional technologies. Even if international markets may be willing to pay a premium for green products in the future, national strategies cannot depend on this, notably if such products are prioritized for domestic consumption.^16^ Gray hydrogen is currently produced at a cost between 0.8 and 5.7 USD/kg_H2_, while green hydrogen from renewable energy sources is estimated at 4–12 USD/kg_H2_, potentially reaching 2 USD/kg_H2_ in certain regions by 2030 under optimistic assumptions.^17^ Energy system models can help track technology improvements and their effect on the competitiveness of different technology options and trade-offs between sectors.^18^ Established frameworks include TIMES, which has been used to create least-cost energy models across multiple spatial scales in more than 40 countries, and all include hydrogen systems to some extent.^18^ The same is true of OSeMOSYS^19^ and PyPSA,^20^ both of which have seen a recent increase in applications in Africa.^20^^,^^21^ Country-level analyses in LMICs are essential for developing national green hydrogen strategies that prioritize local needs, as regional studies often reflect a Eurocentric perspective^22^ or suggest the need for additional studies to guide investments and policy decisions.^23^ National models, by contrast, typically focus on local needs first and consider exports as just one of several options.^24^^,^^25^ Even when aimed at hydrogen production for export, these models can introduce context-specific constraints and assess impacts on the local energy system.^26^^,^^27^

In this work, we present an analysis of Kenya’s national green hydrogen strategy,^28^ expanding a capacity expansion model of the country’s whole energy system.^29^^,^^30^ The model was enhanced to reflect the strategy and incorporates feedback gathered from key local stakeholders during a dedicated workshop. Kenya sees green hydrogen as an opportunity to improve its balance of payments, increase food security and resilience, drive green industrialization, and to attract foreign investments.^28^ Indeed, in recent years, Kenya has attracted the interest of international investors looking to develop hydrogen projects thanks to its vast renewable energy resources, its strategic location in East Africa and well-established international trade links, and interest in the development of clean energy technologies.^31^^,^^32^^,^^33^^,^^34^ Green hydrogen is considered as an option for fertilizer production based on the Haber-Bosch (HB) process^35^ and for green steel production.^36^^,^^37^ According to the national strategy, green hydrogen products are considered for local demand first, and only in a second phase as an option for exports, primarily on the East African market.

To the authors’ knowledge, this study represents the first analysis of green hydrogen potential in a sub-Saharan country using a capacity expansion model. Additionally, the use of a country-level model, combined with the explicit modeling of competition between hydrogen-based and traditional alternatives, enables a detailed assessment of national strategies that prioritize local applications over exports. By leveraging open-source models and data, this study presents a replicable methodology for evaluating hydrogen applications in the agricultural and industrial sectors of other LMICs. Finally, it provides specific, context-driven insights into Kenya’s unique conditions, providing valuable information for local stakeholders involved in implementing the green hydrogen strategy.

## Results and discussion

### Power sources

This study considers three renewable energy sources to power the electrolyzers: solar photovoltaic, wind, and geothermal. To assess the competitiveness of hydrogen-based products against conventional production methods and market alternatives, only dedicated power sources are included. While, under certain grid conditions, strategies utilizing excess electricity from non-dispatchable VRE sources could offer advantages for running electrolyzers, exploring these options falls outside the scope of this study due to the high uncertainty surrounding the future availability of excess electricity on the Kenyan grid.

Solar radiation is uniformly distributed in Kenya as the daily global horizontal irradiance only varies between 5 and 6.5 kWh/m^2^, with minimal differences in the capacity factor profiles and annual productions.^38^ An average profile for the solar photovoltaic power source is hence considered in the current analysis. On the other hand, wind shows significant variations depending on the location, with the highest wind speeds reached in the Lake Turkana region, where Kenya’s largest wind farm is built. Average annual capacity factors can vary between 45% and 66%, with significant differences in the annual profile as well.^38^ For this reason, different wind capacity factor profiles are tested to verify how different wind resources affect the optimal renewable capacity mix, based on the model supply regions (MSRs) defined by Millot et al.^38^ Geothermal is considered to be fully dispatchable and with a capital investment cost aligned with the resources of Olkaria, Menengai, and Suswa fields.^39^

**Figure 1:**
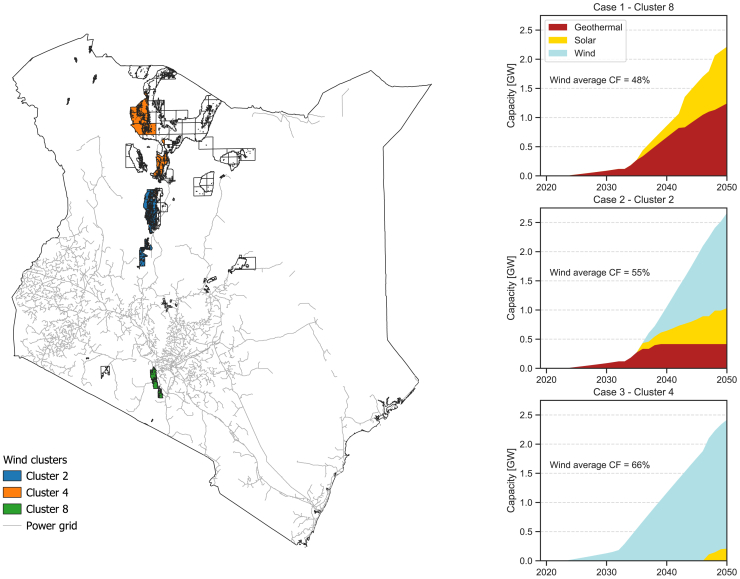
Map of Kenya with relevant clusters highlighted and corresponding capacity mixes for different wind capacity factors Capacity factors are based on clusters of model supply regions (MSRs), as defined in Millot et al.^38^ Case 1 and 3 are, respectively, based on the best and worst MSRs for wind, while case 2 is an intermediate resource.

Figure 1 shows the different capacity mixes required to meet demand from electrolyzers in a reference scenario with significant adoption of hydrogen-based products. Case 1 uses wind capacity factors from the Ngong Hills area, southwest of Nairobi. While this region has the poorest wind resources among the ten areas identified for wind project development in Kenya, it is the closest to regions near Nairobi and Mombasa, where future industrialization is anticipated.^40^ Case 2 is based on a mid-level wind resource location, between Nairobi and Lake Turkana, while case 3 represents the best wind resources, located in the Northern regions around Lake Turkana. The average annual capacity factors for each region and the corresponding wind capacities in 2050 are shown in Table 1. In case 1, no wind capacity is installed, and demand is met through a balanced mix of geothermal and solar. Geothermal is the sole power source until the early 2030s, when solar plants begin to be developed to accommodate the increasing demand for electrolytic hydrogen. Case 2 represents an intermediate scenario where wind complements solar and geothermal. The optimal mix combines all three resources, aligning effectively with the demand profile for hydrogen production. Finally, in case 3, the optimal capacity mix is dominated by wind, which reaches 2.2 GW by 2050, with solar contributing only from the late 2040s onwards.

**Table 1:** Definition of the three power source cases based on different wind model supply regions

Case	MSR^38^	Wind turbine class	Annual average wind turbine CF	Installed wind capacity 2050
1	8	class-3 (ws < 7.5 m/s)	48%	0
2	2	class-2 (7.5 m/s < ws < 8.5 m/s)	55%	1.63 GW
3	4	class-1 (ws > 8.5 m/s)	66%	2.21 GW

For each case, the table summarizes the corresponding model supply region, the class of the wind turbines based on the available wind speeds (ws), the annual average capacity factor (CF) of the wind turbines, and the resulting installed capacity in 2050.

The optimal power mix is therefore highly influenced by location. In the following analyses, we assume a wind capacity factor profile corresponding to case 2. Green hydrogen is seen as a catalyst for industrialization, with plant locations expected to be guided not only by the optimization of power resources but also by factors such as population distribution and logistics. Consequently, this approach considers the potential development of transmission lines to supply power to industrial districts, promoting broader power utilization beyond green hydrogen production. This strategy avoids scenarios where hydrogen is produced in remote locations and transported to industrial regions or where isolated industrial clusters are developed solely for local production of fertilizers and steel.

### Future green hydrogen scenarios

The role of hydrogen in Kenya’s future energy system is analyzed through nine scenarios, created by combining three technological development pathways for electrolyzers (conservative, reference, and optimistic) with three market scenarios for ammonia and steel (low, mid, and high prices). All scenarios are listed in Table 2 and described in greater detail, along with additional considerations on their likelihood, in the Scenarios subsection of STAR Methods.

**Table 2:** Scenarios considered in the analysis

Market\Electr.	Conservative	Reference	Optimistic
Low	S1	S2	S3
Mid	S4	S5	S6
High	S7	S8	S9

Columns represent different levels of technological improvements and cost reductions for water electrolysis technologies, while rows represent different market price levels for international imports and exports of ammonia and steel.

**Figure 2:**
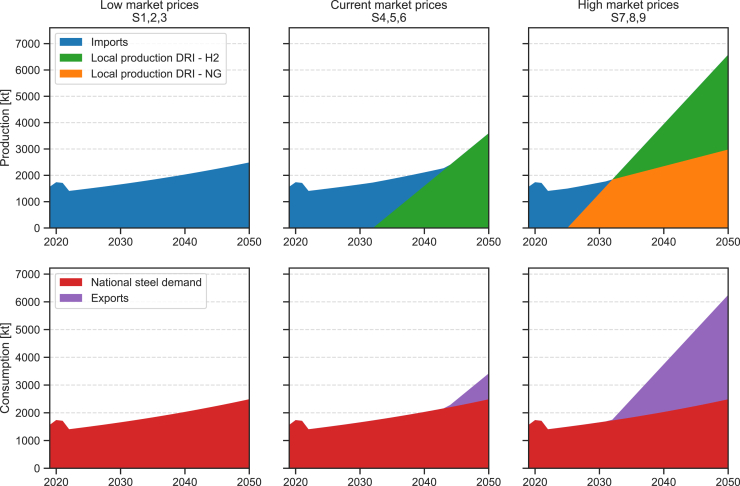
Steel supply sources (top row) and final uses (bottom row) The supply and demand sources are presented for low steel market prices (left column), current market prices (center column), and high market prices (right column). All six plots apply across all electrolyzer development scenarios. For further details, see Figures S1–S3.

In the modeled scenarios, the adoption of green hydrogen for steel production is only dependent on market steel prices, and it is not influenced by the rate of technological development of electrolyzers. Figure 2 hence shows results that can be applied to any scenario of electrolyzer cost reduction and performance improvement. Each column represents steel production and consumption for low, medium, and high market prices. With low steel prices (Figure 2, first column), local production of virgin steel—whether using NG or hydrogen—is not competitive. For NG-based production, this is due to the absence of cheap NG resources and the high costs associated with developing NG import infrastructure. Similarly, in this scenario, green steel is not competitive with steel produced in regions with low-cost NG resources. With steel prices aligned to current market prices (Figure 2, central column), local production based on the direct reduced iron process using natural gas (DRI-NG) remains uncompetitive. However, by 2032, as the constraint on green steel is removed, the cost of alkaline electrolyzers is sufficiently low to make it cost competitive. Production begins to ramp up, constrained only by the growth rate set as an upper limit in the model. By 2050, green steel production covers national demand entirely and can also be in part exported to neighboring countries. Finally, high steel market prices (Figure 2, third column) appear to justify earlier investments in local production using the DRI-NG and electric arc furnace (EAF) processes, with hydrogen-based production ramping up again starting in 2032. By 2032, NG-based production alone could meet national demand, and, by 2050, combined NG and hydrogen-based production could achieve the maximum export level allowed by the model (equivalent to the level of national demand). However, DRI-NG-based production carries the risk of locking into carbon-intensive NG infrastructure, also highlighting the need for further research on the implications of high gas price volatility—such as that recently observed during the Russia-Ukraine crisis.

**Figure 3:**
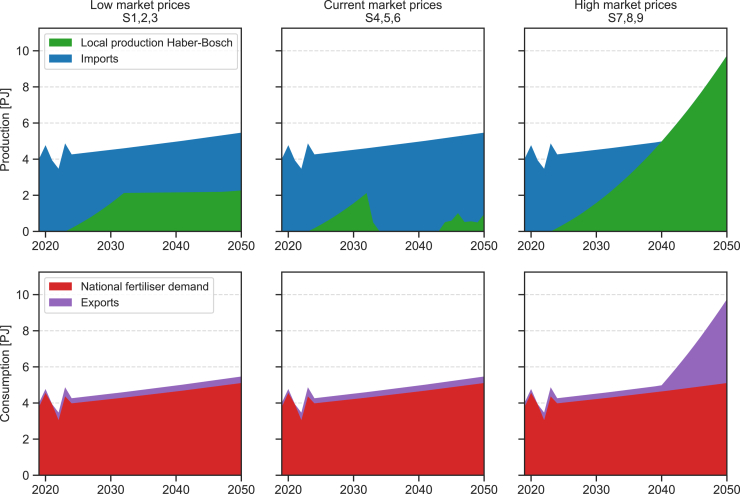
Ammonia for nitrogen-based fertilizer source (top row) and final uses (bottom row) The supply and demand sources are presented for low ammonia market prices (left column), current market prices (center column), and high market prices (right column). All six plots apply across all electrolyzer development scenarios, with differences only on the production side at current market prices. For further details, see Figures S6–S8.

**Figure 4:**
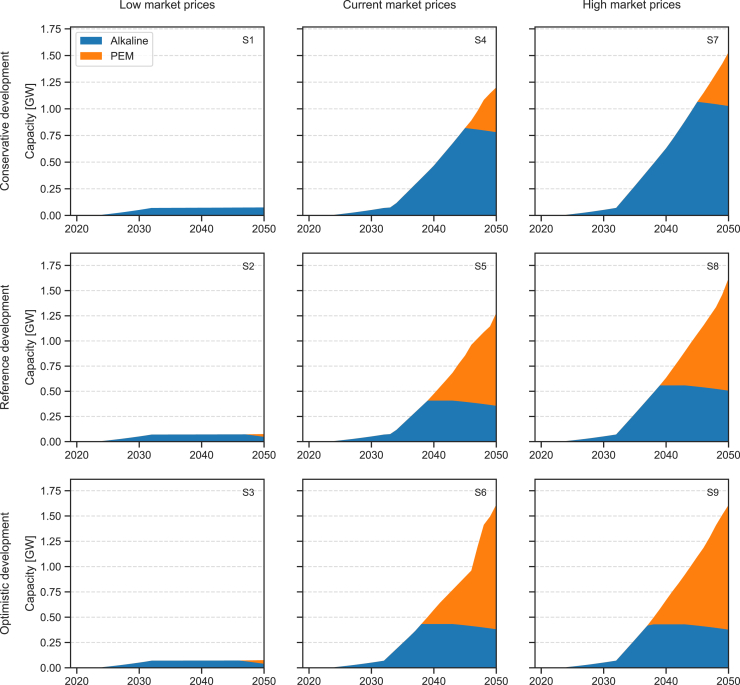
Electrolyzer capacity mix over the nine scenarios simulated Columns represent same market prices, and rows same techno-economic development pathways for the electrolyzers. Corresponding scenario codes are indicated on each subplot.

Under low market prices for ammonia, Kenya’s national strategy faces the risk of domestic production not being cost competitive with imports. As shown in Figure 3, production via the HB process ramps up until 2032, after which it plateaus. This suggests that cost competitiveness is not achieved at any point during the time horizon, and production continues only due to the initial investments driven by the strategy. Similarly, under current market prices, most of the hydrogen capacity developed under the national strategy is redirected toward steel production, as green fertilizers remain uncompetitive, while green steel is economically viable. Only minor quantities of hydrogen are used for ammonia production after 2045, likely to make use of surplus production from VRE power installations driven by steel demand that would otherwise be curtailed. If electrolyzers’ development was not an important determinant for the steel sector, it is a relevant factor for green fertilizers under current market conditions. In an optimistic development scenario, ammonia production, initially sustained by the national strategy, remains relatively stable during the 2030s and early 2040s, before finally ramping up significantly (see center column in Figure S3). This is due to a combination of cheaper electrolyzers, as proton exchange membrane (PEM) becomes cheaper than alkaline ones (Figure 4), and solar and wind cost reductions justifying a relative spike in investments in VRE (see first row, center column, in Figure S3). High market prices once again make technology improvements in electrolysis irrelevant. With ammonia at 1,200 USD/t, there is a steady increase in the local production from green hydrogen, continuing the same path of the national strategy beyond 2032, to completely cover national demand and the current rate of 7% demand exports to neighboring countries by 2040 and reaching 4.6 petajoules (PJ) of exports by 2050.

Figure 4 shows the evolution of the electrolyzer capacity mix through 2050 across the nine scenarios considered. Among the three technology options for water electrolysis, alkaline electrolyzers are the most cost-effective solution for all installations within the time horizon of the national strategy, ending in 2032. Depending on assumptions about electrolyzer development (see Electrolysers and scenarios subsections in STAR Methods for details), the same holds until late 2030s to mid-2040s (see again Figure 4). Beyond that point, PEM electrolyzers become the cost-optimal choice, while solid oxide electrolysis cell (SOEC) electrolyzers are excluded from the mix under all scenarios considered. Market conditions for fertilizers and steel influence hydrogen demand and hence the amount of installed electrolyzing capacity but do not seem to affect significantly the capacity mix. Alkaline and PEM electrolyzers have similar characteristics, and, once PEM electrolyzers become the cheaper option, they gradually dominate the mix, covering new demand and replacing aging alkaline electrolyzers as they reach the end of their operational life. SOEC electrolyzers, due to their significantly higher projected capital cost and efficiency, could potentially serve as a base load technology. However, under the assumptions considered in this study, their investment cost remains too high for inclusion in the mix. This could change depending on the actual trajectories of development of the three technologies, which may not progress in the coordinated manner assumed here. Future studies should consider the relative advancement of these technologies as new data become available.

### Cost of hydrogen

Across the nine simulated scenarios, the resulting levelized cost of hydrogen (LCOH) ranges from 2.7 to 3.7 USD/kg, well within the cost range of other studies considering solar, wind, or geothermal power sources.^41^ These values are calculated over the 2025–2050 period and are based on the actual production of hydrogen determined by the model, representing a weighted average cost of production from 2025 onwards. Due to the gradual adoption of hydrogen-based technologies, the final LCOH values are more heavily influenced by the costs of electrolyzers achieved in the 2040s.

As explained in the power sources subsection, transmission costs have been included in the estimation of the LCOH under the assumption that green hydrogen is produced in industrialized areas, which may be located farther from wind and geothermal resources.

**Figure 5:**
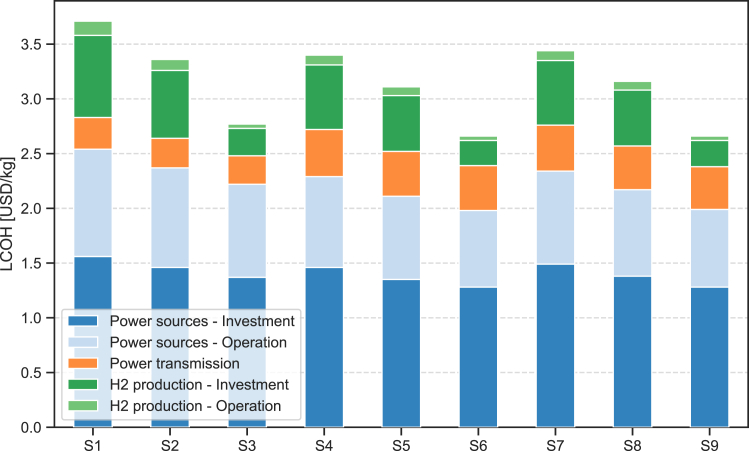
Levelized cost of hydrogen over the nine scenarios simulated The contribution of each price component is shown, distinguishing between investment and operation costs and power and hydrogen generation technologies. Transmission costs are estimated based on an average price for grid expansion. Salvage values at the end of the modeling period are subtracted from power and H_2_ capital costs. Numerical values listed in Table S1.

The obtained prices align with previous studies projecting hydrogen productions costs in Kenya between 1.8 and 3.0 EUR/kg (1.9–3.2 USD/kg at current exchange rates) by 2030.^25^ As shown in Figure 5, the largest share of the LCOH comes from power production, including both capital expenditure and operational costs, the latter predominantly driven by geothermal. The relatively moderate contribution of electrolyzers to the final LCOH explains the limited sensitivity of green fertilizers and steel adoption to development pathways of water electrolysis technologies. This highlights that hydrogen competitiveness depends more on the availability of low-cost renewables and the technological development of hydrogen end use sectors (the latter not being captured in this metric).

In this context, the actual cost of capital for hydrogen projects plays a critical role in determining their cost effectiveness. Robust national strategies that provide clear signals regarding hydrogen demand could reduce uncertainties, lower interest rates, and improve the financial feasibility of such projects.

### Conclusions and policy recommendations

Even when limiting green hydrogen applications to fertilizers and steel, the production of green hydrogen in Kenya could account for 1.5%–23% of the country’s total electricity demand by 2050^39^ across the scenarios explored. This production would be supported by a balanced mix of solar, wind, and geothermal energy, highlighting the potential for renewable resources to drive green hydrogen production and green industrialization.

The cost of hydrogen production is estimated to range between 2.7 and 3.7 USD/kg, with power generation representing the largest component of these costs. These figures place Kenya slightly above the benchmark of 2 USD/kg identified by the IEA as achievable in most promising regions by 2030. It is worth noting that this analysis applied a uniform discount rate of 9%, and actual hydrogen costs could vary depending on project-specific capital costs, emphasizing the importance of context-specific financial conditions.^42^^,^^43^

Electrolyzer costs appear to have only a marginal impact on the cost competitiveness of green hydrogen. Alkaline electrolyzers already show solid techno-economic characteristics, and further improvements are anticipated even under conservative development scenarios. This suggests that the competitiveness of green hydrogen is influenced more significantly by other factors, such as renewable energy costs and broader technological advancements in end use applications.

Beyond hydrogen, a realistic assessment of what establishing fertilizer and steel sectors would entail is needed, considering the associated challenges and opportunities. For nitrogen-based fertilizers, although with some caution, there seems to be potential to capitalize on low-cost hydrogen, making ammonia production competitive. However, the feasibility and specifics of the final step from ammonia to nitrogen-based fertilizers remain unclear. Currently, sub-Saharan Africa has limited fertilizer production, with only five plants producing nitrogen-based fertilizer, three of which are in Nigeria and produce urea.^44^ Recent developments in Kenya’s steel sector indicate ongoing efforts to establish steel production in the country. As in the case of fertilizer, great emphasis should be placed on the extra capital requirements to set up a new industrial sector, including establishing the required logistic chains and building the needed infrastructure. The successful implementation of this phase could then allow to shift the focus toward determining when hydrogen-based production technologies will achieve technological maturity and cost competitiveness with NG. On the latter, this will be important to avoid lock-in to fossil fuel infrastructure and further exposure to volatile international markets. According to our modeling, this transition to green steel production could occur around 2035, contingent on technology spillovers from countries with more advanced steel industries.^14^

While cost competitiveness is a critical factor, other considerations, such as security of supply and industrial development, also play a role in shaping national strategies. Transitioning to domestic production could shift supply dependency from finite products, like fertilizers and steel, to raw materials such as potash for fertilizer production or iron ore for steelmaking. However, (1) raw materials may have stronger or more accessible markets, including local ones, compared to finished products, and (2) strategic decisions could enhance Kenya’s self-reliance. For example, developing local iron ore production or increasing the share of urea in total fertilizer output could reduce dependence on imports and strengthen industrial capacity. Balancing these factors with cost considerations could position Kenya to achieve greater supply chain resilience while fostering long-term economic and industrial growth.

The competitiveness of locally produced fertilizer and steel in regional markets will also depend on regional competition and trade policies. While this aspect is beyond the scope of the present study, it is a critical consideration for policymakers. As discussed in the introduction, international markets for green products should not be the primary focus of national strategies if local applications are to be prioritized. However, once established, such markets could help mitigate the risks associated with competition in local and regional markets.

Establishing green hydrogen-based industries requires careful consideration of broader capital requirements and financing barriers, extending beyond the shift to hydrogen itself analyzed in this study. Prioritizing investments that are less reliant on the development of new international markets, while considering co-benefits such as supply security and industrial development, will be key to ensure sustainable growth. Actionable recommendations include (1) sending clear policy signals to attract investors and lower the cost of capital; (2) encouraging and incentivizing financing for low-risk early green fertilizer projects; (3) developing a fertilizer industry capable of leveraging green hydrogen-derived ammonia as it becomes available; and (4) strategically planning the growth of the steel sector, balancing the risk of lock-in to established processes with opportunities presented by emerging technologies.

Capacity expansion models that integrate hydrogen technologies can be valuable tools for testing national green hydrogen strategies from a single-country perspective, prioritizing local applications, but with consideration of external factors. The Kenya case study, developed using an open-source model, provides insights that could be highly relevant for other LMICs as they design and implement their own national strategies. As with all modeling studies, contextualization to account for a range of considerations outside of the model is critical, including financing, the investment environment, regulatory frameworks, and supply chains.

### Limitations of the study

This study comes with a set of limitations and opportunities for future work.

First, water consumption during the electrolysis stage has been neglected. Evaluating its potential impact on Kenya’s water resources—considering plant siting and low precipitation years—could be of interest of the implementation phase of the national strategy. Nexus frameworks that integrate water systems and land using capacity expansion models could leverage on the model developed for this study to integrate such considerations.^45^

Second, capacity expansion models inherently have limitations in representing the operational dynamics of the power sector.^39^ While this study highlights the trend of building dedicated power sources for hydrogen production, future work could explore different production layouts and assess the grid impact of large-scale hydrogen production in greater detail.

Finally, this study employs a scenario-based approach to analyze green hydrogen futures in Kenya, relying on a set of assumptions to limit the number of scenarios and maintain clarity. While this provides useful insights, a more systematic approach to uncertainty could increase the robustness of our findings. Methods for decision-making under deep uncertainty, like robust decision-making,^46^ could be applied to further explore the influence of uncertain parameters across scenarios, including the critical role of the cost of capital for different technologies.^42^^,^^43^

## Resource availability

### Lead contact

Further information and requests for resources and materials should be directed to and will be fulfilled by the lead contact, Pietro Lubello (p.lubello@ucl.ac.uk).

### Materials availability

This study did not generate new materials.

### Data and code availability

All data used in this study are obtained from publicly available sources and are listed in the manuscript or in the supplementary information. The OSeMOSYS model and data files used in this study are available on Zenodo: https://doi.org/10.5281/zenodo.15056038.

## Acknowledgments

This material has been produced with support from the 10.13039/100004918Climate Compatible Growth (CCG) program, which brings together leading research organizations and is led out of the STEER center, Loughborough University. CCG is funded by UK aid from the UK government. However, the views expressed herein do not necessarily reflect the UK government’s official policies.

J.O. acknowledges the support of the 10.13039/501100011099Deutsche Gesellschaft für Internationale Zusammenarbeit (GIZ) GmbH. The views expressed are those of the authors and do not necessarily reflect the views of GIZ or its partners.

The authors would also like to thank Vignesh Sridharan for his insightful feedback on the methodology and presentation of results.

## Author contributions

J.O., P.L., and S.P. conceived the study and developed the hydrogen implementation in the model. J.O. and P.L. worked on the modeling of the scenarios, and M.A., K.M., and M.M. supported the calibration of the model to the Kenyan context. All authors contributed to the drafting and reviewing of the manuscript.

## Declaration of interests

The authors declare no competing interests.

## STAR★Methods

### Key resources table

**Table:** 

REAGENT or RESOURCE	SOURCE	IDENTIFIER
**Deposited data**		

Kenya WESM with GH_2_ implementation	This paper	https://doi.org/10.5281/zenodo.15056038
Wind clusters based on model “supply regions”	Sterl et al.^47^	https://doi.org/10.1038/s41597-022-01786-5

**Software and algorithms**		

OSeMOSYS	Howells et al.^19^	https://doi.org/10.1016/j.enpol.2011.06.033

### Method details

#### Kenya OSeMOSYS Whole Energy System Model (WESM)

The model used in this study is a representation of Kenya’s Whole Energy System Model (WESM), which uses the OSeMOSYS modeling framework.^19^ Primary energy can be sourced either through imports or local production. Locally available renewable energy sources feed into the power system, and the electricity generated is then distributed to end-use sectors. The model provides a detailed description of the power sector, based on the Kenya OSeMOSYS Power Sector Model.^39^ Bioenergy can be directed either to the power sector, to be fired in biomass power plants, or to the residential sector, for cooking and heating water in the form of fuel wood, charcoal, bioethanol or biomethane. Fossil fuels are exclusively supplied through imports and distributed to end use sectors and the power system, with no domestic production or refining capacity considered. The end-use sectors represented in the energy system include the agricultural, commercial, industrial, residential, and transport sectors. Demands are modeled as energy service demands, enabling the model to capture potential efficiency gains and emissions reductions through fuel substitution across a range of technology options in different sectors.

End-use sectors vary in the level of detail at which they are represented, primarily depending on data availability and the specific focus of the modeling. The agricultural, industrial, and commercial sectors are relatively simpler, whereas a broader range of end-use technologies and fuels is considered in the residential and transport sectors. An overview of each sector is provided in section *Kenya Whole Energy System Model end use sectors* of the *Supplemental information*, while a comprehensive description of the model and its complete reference energy system can be found in the model’s documentation.^30^

Energy service demand (ESD) projections are estimated off-model and provided as an input.^48^ Starting from current final energy demand levels and the existing technology mix, ESDs are calculated for each sector. The annual energy use, expressed in petajoules (PJ) and associated with various technologies providing the same service, is multiplied by each technology’s efficiency, expressed in energy service per PJ (e.g., bvkm/PJ for passenger vehicles). The total of these values represents the energy service demand for the reference year for the service in question. To project demand toward 2050, common drivers are considered, including population, urbanisation rate, urban and rural household occupancy, household electricity use, Gross Domestic Product (GDP), and Gross Value Added (GVA), depending on the sector.^48^

Beyond the addition of hydrogen-related supply chains, detailed in the following subsections, an additional modification has been introduced to the model. To more accurately represent future expenses associated with adopting natural gas in Kenya, the model explicitly includes the cost of building LNG import infrastructure, following the approach outlined by Millot et al.^38^ Import capacity is restricted to zero until 2030. From 2030 onward, the maximum annual import capacity increases by 1 Mt/year every five years, reaching a total of 5 Mt/year by 2050. The investment includes a cost of 570 MUSD per Mt/year for the regasification terminal and 300 MUSD for the domestic pipeline, totalling approximately 29 MUSD/PJ/year for natural gas import infrastructure capacity.

#### Kenya OSeMOSYS WESM end use sectors

In this section, we briefly describe the end use sectors included in the Kenya Whole Energy System Model.

Agriculture – The only demand type considered for the agricultural sector is a generic demand type, obtained from the IEA energy balances^49^ and representing fuel consumption to operate the agricultural machinery, expressed in useful energy terms. There is one technology option per fuel type, and fuels considered are diesel, gasoline, and heavy fuel oil.

Commercial sector – A single demand is considered for the commercial sector, and it represents the consumption of electricity for a variety of services in buildings and facilities not classified as residential, industrial, or agricultural. This includes, for example, electricity used for lighting and operating equipment in offices, retail stores, schools, hospitals, hotels, and other public and service-oriented buildings, but it does not consider low temperature heat production, as it is null in the energy balance of ref. 49. A single generic technology with unit efficiency and using electricity as fuel is considered in the model.

Industry – The industrial sector is slightly more complex than the first two. Three different types of demand are considered, as obtained from IEA energy balances^49^: non-metals and cement, food processing, and other processes. The demand for the non-metals and cement subsector can only be covered by technologies using coal as fuel. Similarly, only electricity is considered as an option for the food processing subsector. The other processes subsector includes several different processes, including steel production. Technologies considered are based on various fuels inputs, including coal, electricity, diesel, heavy fuel oil and kerosene.

Residential sector – The residential sector is the most complex sector included in the model, as it also represents the highest share of the final energy consumption in the country. Demands are divided between lighting, cooling, cooking and other. Demand levels are originally obtained from the IEA,^49^ and have been updated based on data provided by Nuvoni Center for Innovation Research and the Modern Energy Cooking Services program.^29^ Each demand is split between urban and rural areas, to account for the significant differences in the two areas. Cooling and other demands can only be satisfied by technologies using electricity as an input fuel. Lighting options include both electricity and kerosene. Finally, the cooking sector offers numerous technology options, including different ones for the same type of fuel. For example, e-cooking technologies considered are coil, induction, and electric pressure cookers, while wood stoves can be either traditional or improved, as in the case of charcoal. A complete description can be found in the model’s documentation.^30^

Transport sector – Transports include national aviation and shipping, the railway system and road transport. The latter is divided between buses, cars, freight, light commercial vehicles, and two- and three-wheelers. Each of the subsector of road transport has three technology options, namely diesel, gasoline, and electricity, except for freight transport, where only diesel and gasoline are considered. Aviation demand can only be satisfied by technologies using jet fuel, shipping by technologies using heavy fuel oil, and electric and diesel trains are considered. Demands are obtained from the IEA energy balances^49^ and the Transport Inventory and Greenhouse Gas Emissions Reporting (TrIGGER) tool.^50^

#### Nitrogen-based fertiliser and steel sector representation in the model

This study considers the production of green hydrogen only through dedicated power plants. Based on Kenya’s natural resources, solar, wind and geothermal energy are considered as options for power generation. Electricity is then fed to the electrolysers, with PEM, alkaline (ALK), and solid oxide electrolysis cell (SOEC) technologies considered as options. As shown in Figure S4, for hydrogen used in fertiliser production, electricity is also supplied to the Haber-Bosch process, where hydrogen is converted to ammonia. Locally produced ammonia can be used to meet domestic demand for nitrogen-based fertilisers or to be exported. Similarly, imported ammonia can be directed toward local demand or re-exported. However, export prices are set at 99% of import prices to avoid imports solely for regional re-export, as this fall outside the scope of the present work. As explained in the subsection *Nitrogen-based fertilisers*, demand for nitrogen-based fertilisers is represented in terms of ammonia equivalent demand, without accounting for the subsequent steps in final product manufacturing.^12^

In the case of hydrogen for green steel production (see Figure S5), renewable electricity is also fed to the electric arc furnace (EAF). The furnace is set to work with 5% scrap metal and 95% sponge iron,^17^ produced through direct reduced iron (DRI) processes based either on hydrogen or natural gas. The steel produced by the EAF can then be used to cover local demand or for exports. As in the case of ammonia, export prices for steel are set to 99% of import ones, to avoid imports to be considered for regional re-export.

#### Renewable energy sources for green hydrogen

Three renewable power sources are considered to feed the electrolysers: solar, wind, and geothermal. Capital cost projections for solar and wind are based on the Net Zero scenario of the IEA’s World Energy Outlook (WEO),^51^ while for geothermal a constant investment cost based on current prices is considered and set to 3800 USD/kW.^39^ The capacity factor of solar power plants is based on a national average, as solar resources are quite evenly spread through the country.^38^ On the other hand, wind capacity factors have much more significant variations, with average annual capacity factors between 45% and 66%.^38^ Three different capacity factor profiles have been considered based on Model Supply Regions (MSRs) defined in Millot et al.^38^.

(1) Case 1 (MSR 8) – Ngong Hills area, southwest of Nairobi. Lowest wind speeds among the ten best areas for wind development in Kenya. It is the closest region to Nairobi and Mombasa.
(2) Case 2 (MSR 2) – Intermediate level of wind resources in a region relatively close to Nairobi and halfway between Nairobi and the high wind regions around Lake Turkana.
(3) Case 3 (MSR 4) – Region containing the best onshore wind resources in Kenya. This is where the Lake Turkana wind farm is located.

The dataset used to characterise the wind regions is openly available and contains data for all African countries.^47^

#### Electrolysers

Three technology options are considered to produce green hydrogen from renewable electricity: PEM, alkaline (ALK), and solid oxide electrolysis cell (SOEC). Each technology uses electricity to turn water into hydrogen and oxygen. Water consumption and oxygen co-production are not accounted for in this work. The parameters needed for the techno-economic characterisation of each technology are obtained from a brief literature review of peer-reviewed and gray literature. Given the high levels of uncertainty around cost and performance evolution in the future, three different scenarios of development are considered – conservative, reference and optimistic. A summary of projected capital cost, efficiency, and stack lifetime ranges obtained from the literature review are listed in Tables S2–S4.

Based on the data collected, the three technology development scenarios used in this study are defined (see *Scenarios* subsection). Resulting costs, efficiencies and operational lifetimes of the stacks for available years are summarised in Tables S5–S7, while input parameters for the missing years are obtained by linear interpolation of tabulated values, as shown in Figure S4.

Operational costs are considered as fixed annual costs and set as 2.5% of investment costs, regardless of the type of electrolyser. This is in line with all sources considered, that set operating costs between 1 and 3%, without any significant distinction between the different technologies. Similarly, the availability factor is set to 98%.^52^

In OSeMOSYS, the operational life parameter is constant over the entire time horizon considered for all technologies. In this study, the operational life of each electrolyser is set to 20 years. However, to represent the expected technological improvements in the stack lifetime and the significant differences between alkaline, PEM and SOEC technologies (see Table S7), the capital cost of each electrolyser is increased by adding the discounted cost of N substitutions of the stack, as shown in Equation 1.

(Equation 1) $$C_{inc}=C+\sum_{i=1}^{N}\frac{C/4}{{\left(1+r\right)}^{\mathrm{int}\left(i\cdot l/N\right)}}$$

Where C_inc_ is the increased capital cost of the electrolyser, C the original capital cost, r the discount rate and l the lifetime of the stack. Based on the literature data summarised in Table S7, and averaging between current performances and 2050 projections, one substitution is considered for alkaline and PEM electrolysers (based on an average stack lifetime of 10 years), and three for solid-oxide ones (based on an average stack lifetime of 5 years).

#### Nitrogen-based fertilisers

Historical data on fertiliser consumption and imports in Kenya is openly available through the AfricaFertilizer initiative’s online data platform, which offers data and market analysis on fertilisers in sub-Saharan Africa.^53^ Figure S5 shows the annual apparent consumption and imports of different types of fertilisers for the period 2012–2023.

Green hydrogen can be used to produce ammonia via the Haber-Bosch process, which is then utilised in nitrogen-based fertiliser production. To estimate the current potential demand for ammonia, we first determine nitrogen demand based on the fertiliser mix and the nitrogen content of each type. We then calculate the equivalent amount of ammonia required to meet this nitrogen demand, applying an 82% conversion rate based on ammonia’s nitrogen mass content of 0.82 kg_N_/kg_NH3_. Typical nitrogen content values for the most common fertilisers are provided in Table S8.

Inferred nitrogen consumption and imports are thus calculated, with exports determined as the difference between the two trends (Figure S6). For 2021, export levels are set to zero, as consumption exceeds imports, likely due to part of the demand being met by existing fertiliser stocks. On average, exports account for 7.7% of annual demand. Based on ammonia’s nitrogen content, demand for ammonia in the base year (2019) is set to 201.4 kt. Fertiliser demand projections are based on the Food and Agriculture Organization (FAO) estimates of 9% growth over the period 2023–2032 for Sub-Saharan Africa,^54^ which have been extrapolated out to 2050 (see Figure S7).

All relevant techno-economic parameters required to characterise the Haber-Bosch process are listed in Table S9. The hydrogen storage necessary to ensure smooth operation is included in the HB investment costs and is estimated to range between 3.3 and 4.9 USD/MWh_H2_.^55^

#### Steel sector

Over the past decade, steel demand in Kenya has fluctuated between approximately 1000 kt/y and 1800 kt/y, without showing a clear trend^56^^,^^57^ (see also Table S10). This demand is met entirely through imports, with negligible re-exports and minimal local production, primarily from a few minor induction furnaces processing scrap steel. However, a major new plant recently opened in Kwale County, with plans to ramp up production from scrap steel through electric arc furnace (EAF) in the coming years, and eventually start producing virgin steel through direct reduced iron (DRI) process as well.^58^ Given the high uncertainty for steel demand in Kenya, a conservative rate of 2% year-on-year is considered toward 2050 based in recent global trends^59^ (see Figure S8).

Table S11 lists all cost assumptions considered for the EAF and DRIs (natural gas and hydrogen) processes, while Table S12 reports the efficiencies of the process with respect to different commodities. The share of scrap steel for the EAF process is considered to be between 0% and 5%,^37^^,^^60^ and hence omitted. Iron ore (58% Fe) unit cost is set to 60 USD/t.

#### Scenarios

Scenarios analyzed are based on the combination of different assumption around the techno-economic development of water electrolysis technologies and market prices for ammonia and steel, as shown in Table 2.

Electrolysers’ technological improvements are translated into different projections for cost reductions and efficiency gains, corresponding to a conservative, reference and optimistic development scenarios (see also *Electrolysers* subsection). Given the lack of detail in the representation of operational life for technologies in OSeMOSYS, no variation in the lifetime of the electrolysers is introduced, but a generic value of 20 years is considered for all three technologies. Differences in the cell stack lifetimes are reflected in an increase in capital cost, as described in the *Electrolysers* subsection.

For the purposes of scenario formulation, market prices for ammonia and steel, though only loosely related, are assumed to follow similar trend. This results in three scenarios corresponding to low, mid and high market prices, based on historical prices over the last ten years and listed in Table S13.

All scenarios are built around the assumption that the initial uptake of green hydrogen in Kenya follows the national green hydrogen strategy.^28^ Based on the strategy, a target of 20% of national nitrogen-based fertiliser demand covered by green hydrogen-based fertilisers is set for 2027 and 50% by 2032, and no commercial production of green steel. After 2032, the model is free to choose the cost optimal solution, and the only constraints are set around the rate of potential uptake of new technologies. The production of ammonia for fertiliser is bounded by the expansion of the Haber- Bosch process, that continues to follow the trajectory set by the national strategy to surpass national demand by 2040 and reach almost twice the domestic demand level by 2050. Additionally, from 2024 exports are bounded by the most stringent constraint between (i) an annual increase of 10% of national demand, starting from current export levels or (ii) exports being equal to annual demand (see Figure S9 for details). Exports have a lower bound as well, set to the current level of 7% of the annual demand. The steel sector is constrained by an upper production limit for steel produced via electric arc furnaces, beginning in 2026 and increasing by 250 t/y. Similarly to the fertiliser sector, exports are limited either by the 10% of the domestic demand annual increase starting from 2024 levels, or by a higher limit set to be equal to national demand (see Figure S10).

Finally, while all scenarios are presented as equally plausible, their actual likelihood could be explored further. The high uncertainty surrounding the evolution of electrolyser technologies makes it challenging to assign clear probabilities to each pathway. However, market trends for ammonia and steel could be more easily investigated. For example, in the case of steel, projected oversupply suggests that market prices are likely to remain low to current levels in the short to medium term.^59^

#### Levelized cost of hydrogen

The levelized cost of hydrogen (LCOH) can provide additional insights on the key cost components influencing hydrogen production prices. This is particularly relevant for this study, as it not only considers electricity prices but also compares different power mixes.

The LCOH is calculated retrospectively, based on the results from the OSeMOSYS runs for the various scenarios. It is determined by dividing the discounted total investment and operational costs for the dedicated renewable energy capacity and electrolysers over the analysis period (2025–2050) by the discounted total hydrogen produced during the same time frame. Any discounted salvage value of investments extending beyond 2050 is subtracted from the total costs as obtained from OSeMOSYS. The resulting expression is:

(Equation 2) $$LCOH=\frac{\sum_{t}\sum_{i}\frac{{CAPEX}_{i,t}\;+\;{OPEX}_{i,t}}{{\left(1\;+\;r\right)}^{t}}-S_{i}}{\sum_{t}\frac{H_{t}}{{\left(1\;+\;r\right)}^{t}}}$$

Where *CAPEX* represents the capital expenditures and *OPEX* the operational ones, *S* is the discounted salvage value at the end of the modeling period, *H* the annual hydrogen production, *r* the discount rate, and *i* and *t* the indexes to sum over technologies and time. The technologies considered are solar, wind and geothermal for power production, and alkaline, PEM and SOEC electrolysers for hydrogen production.

### Quantification and statistical analysis

There are no quantification or statistical analyses to include in this study.

## Supplemental information

Document S1. Figures S1–S12 and Tables S1–S13

Table S14. Full list of fertilizers considered in the study and corresponding nitrogen content expressed on a mass basis
