# Supplementary material for: Green hydrogen futures in LMICs: Opportunities for fertilizer and steel production in Kenya
Source: iScience. 2025 Mar 27;28(4):112298. doi: 10.1016/j.isci.2025.112298 (PMC12005298; doi:10.1016/j.isci.2025.112298)
Supplement: Document S1. Figures S1–S12 and Tables S1–S13 [file mmc1.pdf]

iScience, Volume *28*

Supplemental information

Green hydrogen futures in LMICs: Opportunities for fertilizer and steel production in Kenya

Pietro Lubello, Joshua Oduor, Anne Nganga, Martin Mutembei, Francis Njoka, Michelle Akute, Kihara Mungai, and Steve Pye

Supplemental Tables and Figures

Tables – Modelling results

Table 1 Levelized cost of hydrogen (LCOH) over the nine scenarios simulated.

	LCOH [USD/kg] 2025-2050
**S1**	3.70
**S2**	3.36
**S3**	2.77
**S4**	3.42
**S5**	3.12
**S6**	2.67
**S7**	3.45
**S8**	3.16
**S9**	2.66

Tables – Modelling assumptions

Table S2 Summary of the capital costs for alkaline, proton exchange membrane (PEM) and solid oxide electrolysis cells (SOEC) electrolysers. Current costs are listed, as well as projections for 2030 and 2050 and the respective sources. Where intervals are given, typical values are shown in brackets. IEA values are given for a generic water electrolysis technology. All values are expressed in USD/kW, reference year 2020.

Technology	Current (2020-2024)	2030	2050	Source	Year
Water electrolysis	China 1000<br>Global 1750	China 400<br>Global 440	320-340	IEA[S1]	2021
Water electrolysis	China 1070<br>Global 1640	China 420<br>Global 610	-	IEA[S2]	2023
Water electrolysis	China 1100<br>Global 2160	China 620<br>Global 960	-	IEA[S3]	2024
Alkaline (10 MW)	1525-2275 (1900)	875	375-475 (475)	DEA[S4]	2024
Alkaline (100 MW)	950-1450 (1200)	550	250-350 (300)	DEA[S4]	2024
Alkaline (1 GW)	875-1325 (1100)	500	225-325 (275)	DEA[S4]	2024
PEM (10 MW)	1525-2275 (1900)	950	400-600 (500)	DEA[S4]	2024
PEM (100 MW)	1050-1550 (1300)	650	275-425 (350)	DEA[S4]	2024
PEM (1 GW)	950-1450 (1200)	600	250-400 (325)	DEA[S4]	2024
SOEC (1 MW)	3200-4800 (4000)	1725	1450	DEA[S4]	2024
SOEC (10 MW)	2325-3475 (2900)	1250	1050	DEA[S4]	2024
SOEC (100 MW)	1450-2150 (1800)	775	650	DEA[S4]	2024
PEM	900-1800	650-1400	400-1000	Blanco et al.[S5]	2018
SOEC	785	450	300	Blanco et al.[S5]	2018
Alkaline	437-1500 (700)	357-800 (621)	-	ENTSO-E[S6]	2022
PEM	613-2000 (1160)	350-1350 (663)	-	ENTSO-E[S6]	2022
SOEC	2520-5040 (3083)	715-2500 (1706)	-	ENTSO-E[S6]	2022
Alkaline	500-1000	-	200	IRENA[S7]	2020
PEM	700-1400	-	200	IRENA[S7]	2020
SOEC	2000 (stack)	-	300	IRENA[S7]	2020

Table S3 Summary of the efficiencies of alkaline, proton exchange membrane (PEM) and solid oxide electrolysis cells (SOEC) electrolysers. Current efficiencies are listed, as well as projections for 2030 and 2050 and the respective sources. Where intervals are given, typical values are shown in brackets. IEA values are given for a generic water electrolysis technology. All values are expressed as unit fractions, unless otherwise specified.

Technology	Current (2020-2024)	2030	2050	Source	Year
Water electrolysis	0.64	0.69	0.74	IEA [S1]	2021
Water electrolysis	0.65	0.69	-	IEA [S2]	2023
Water electrolysis	0.66	0.69	-	IEA[S3]	2024
Alkaline	0.65	0.75	0.769	DEA[S8,S9]	2021
PEM	0.6	0.8	0.9	DEA[S8,S9]	2021
SOEC	0.74	0.84	0.9	DEA[S8,S9]	2021
PEM	0.65-0.75	0.7-0.8	0.75-0.86	Blanco et al.[S5]	2018
SOEC	0.905	0.949	0.949	Blanco et al.[S5]	2018
Alkaline	0.63-0.70	0.65-0.71	-	ENTSO-E[S6]	2022
PEM	0.61-0.70	0.63-0.75	-	ENTSO-E[S6]	2022
SOEC	0.74-0.81	0.77-0.88	-	ENTSO-E[S6]	2022
Alkaline	0.50-0.68<br>50-78 kWh/kg_{H2}	-	> 0.70<br>< 45 kWh/kg_{H2}	IRENA[S7]	2020
PEM	0.50-0.68<br>50-83 kWh/kg_{H2}	-	> 0.80<br>< 45 kWh/kg_{H2}	IRENA[S7]	2020
SOEC	0.75-0.85<br>40-50 kWh/kg_{H2}	-	> 0.85<br>< 40 kWh/kg_{H2}	IRENA[S7]	2020

Table S4 Summary of the stack lifetime of alkaline, proton exchange membrane (PEM) and solid oxide electrolysis cells (SOEC) electrolysers. Current operational lifespans are listed, as well as projections for 2030 and 2050 and the respective sources. IEA values are given for a generic water electrolysis technology. Values can be expressed in terms of thousands of working hours or years of operation.

Technology	Current (2020-2024)	2030	2050	Unit	Source	
Water electrolysis	50	50	50	khours	IEA[S1]	2021
Water electrolysis	50	50	-	khours	IEA[S2]	2023
Water electrolysis	50	50	-	khours	IEA[S3]	2024
Alkaline	30	30	30	years	DEA[S8,S9]	2021
PEM	30	30	30	years	DEA[S8,S9]	2021
SOEC	30	30	30	years	DEA[S8,S9]	2021
PEM	35-60	40-80	50-100	khours	Blanco et al.[S5]	2018
SOEC	2	10	20	years	Blanco et al.[S5]	2018
Alkaline	60-75	90-100	-	khours	ENTSO-E[S6]	2022
PEM	50-80	60-90	-	khours	ENTSO-E[S6]	2022
SOEC	10.0-20	40-60	-	khours	ENTSO-E[S6]	2022
Alkaline	60	-	100	khours	IRENA[S7]	2020
PEM	50-80	-	100-120	khours	IRENA[S7]	2020
SOEC	< 20	-	80	khours	IRENA[S7]	2020

TableS5 Capital costs for alkaline, proton exchange membrane (PEM) and solid oxide electrolysis cells (SOEC) electrolysers considered in this study, based on the values listed in Table S2. All values are given in USD/kW, reference year 2020.

	**Conservative**				
	**2020**	**2025**	**2030**	**2040**	**2050**
ALK	2325	2000	1675	1010	350
PEM	2825	2500	2175	1300	425
SOEC	5425	4600	3775	2365	950
	**Reference**				
	**2020**	**2025**	**2030**	**2040**	**2050**
ALK	1995	1670	1345	820	300
PEM	2410	2090	1765	1060	350
SOEC	4700	3875	3050	1925	800
	**Optimistic**				
	**2020**	**2025**	**2030**	**2040**	**2050**
ALK	1075	750	425	340	250
PEM	1625	1300	975	625	275
SOEC	3975	3145	2320	1485	650

Table S6 Efficiencies for alkaline, proton exchange membrane (PEM) and solid oxide electrolysis cells (SOEC) electrolysers considered in this study, based on the values listed in Table S3. All values are expressed as unit fractions.

**Technology**	**Conservative**			**Reference**			**Optimistic**		
	**2020**	**2030**	**2050**	**2020**	**2030**	**2050**	**2020**	**2030**	**2050**
ALK	0.5	0.65	0.7	0.63	0.7	0.73	0.68	0.75	0.77
PEM	0.5	0.63	0.74	0.65	0.7	0.8	0.75	0.8	0.86
SOEC	0.74	0.84	0.85	0.8	0.89	0.9	0.91	0.95	0.95

Table S7 Cell stack operational life for alkaline, proton exchange membrane (PEM) and solid oxide electrolysis cells (SOEC) electrolysers considered in this study, based on the values listed in Table S4. All values are given in years considering 8000 h/y of operation, or around 90% capacity factor.

**Technology**	**Conservative**			**Reference**			**Optimistic**		
	**2020**	**2030**	**2050**	**2020**	**2030**	**2050**	**2020**	**2030**	**2050**
ALK	8	11	13	9	12	13	10	13	13
PEM	4	5	6	7	8	11	10	11	15
SOEC	1	5	10	1	6	10	2	7.5	10

Table S8 Nitrogen content, on a mass basis, per type of fertilizer. NPK fertilisers contain various blends of nitrogen, phosphorus and potassium, with nitrogen content ranging anywhere from 1% to 40%. Remaining fertilisers have been split between Others (no nitrogen content) and Others H_2 (some nitrogen content that could be produced through H_2). The full list of fertilisers and their corresponding nitrogen content is given in Table S14.

**Fertiliser**	**N content [%]**
Calcium Ammonium Nitrate	27
Calcium Nitrate	15.5
Diammonium Phosphate	18
Muriate of Potash	0
NPK	1- 40
Others	0
Others H_2	1- 40
Urea	46

Table S9 Techno-economic parameters for the characterization of the Haber-Bosch process. Investment costs include hydrogen storage at 3.3-4.9 USD/MWh$_{H2}$ to achieve 8000 h/y utilization rate [S2]. Most of the energy needed for the process is provided by the exothermic reaction, electricity is needed to power motors, heat exchangers and pressure and temperature control equipment [S10].

**Parameter**	**Value**	**Source**
Efficiency (H_2 to NH_3)	0.98	[S10]
Electricity consumption	2.2 GJ/t$_{NH3}$	[S2]
Availability factor	0.95	[S2]
Capital cost	770 USD/kW	[S2]
Fixed cost	23.1 USD/kW/y	[S2]

Table S10 Steel demand in Kenya for the years 2012 to 2022. Data obtained from World Steel Association[S11] *and Kenya National Bureau of Statistics*[S12].

Year	Demand [kt/y]	Source
2012	1028	[S11]
2013	1409	[S11]
2014	1436	[S11]
2015	1835	[S11]
2016	1608	[S11]
2017	1465	[S11]
2018	1519	[S11]
2019	1564	[S12]
2020	1738	[S12]
2021	1711	[S12]
2022	1406	[S12]

Table S11 Investment and operating costs for direct reduced iron (DRI) and electric arc furnace (EAF) technologies. DRI can be either natural gas- or hydrogen-based. Capital costs are obtained from Rosner et al.[S13]*, and checked against the latest available data from IEA Global Hydrogen Review* [S1]*. Operating costs are only considered as annual fixed costs and set to 7.5% of the capital costs* [S1].

Technology	Capital cost [MUSD/kt/y]	Fixed cost [MUSD/kt/y]	Variable cost [MUSD/kt]
DRI-NG	0.750	0.056	0
DRI-H2	0.597	0.045	0
EAF	0.234	0.018	0

Table S12 Energy and materials consumption for the steel making processes. Values are obtained for a direct reduced iron (DRI) and electric arc furnace (EAF) integrated processes as illustrated in Rosner et al. [S13]*, under the assumption that the EAF is working with a 100% sponge iron input and no scrap steel. The latter hypothesis is also well aligned with the IEA's assumption of 5% scrap steel* [S1].

Technology	Commodity	Consumption
DRI-NG	Natural gas	10.8 GJ/$t_{sponge\ iron}$
DRI-NG	Iron ore	1.54 t/ $t_{sponge\ iron}$
DRI-H2	Hydrogen	9.1 GJ/ $t_{sponge\ iron}$
DRI-H2	Iron ore	1.54 t/ $t_{sponge\ iron}$
EAF	Electricity	2 GJ/t_{steel}
EAF	Sponge iron	1.05 t/t_{steel}

Table S13 Market prices for ammonia and steel in low, current and high market prices scenarios. Sources are listed in the Table.

Commodity	Low	Mid	High	Source
**Ammonia [USD/t]**	230	450	1200	[S14]
**Steel [USD/t]**	300	800	2000	[S15]

Figures – Modelling results

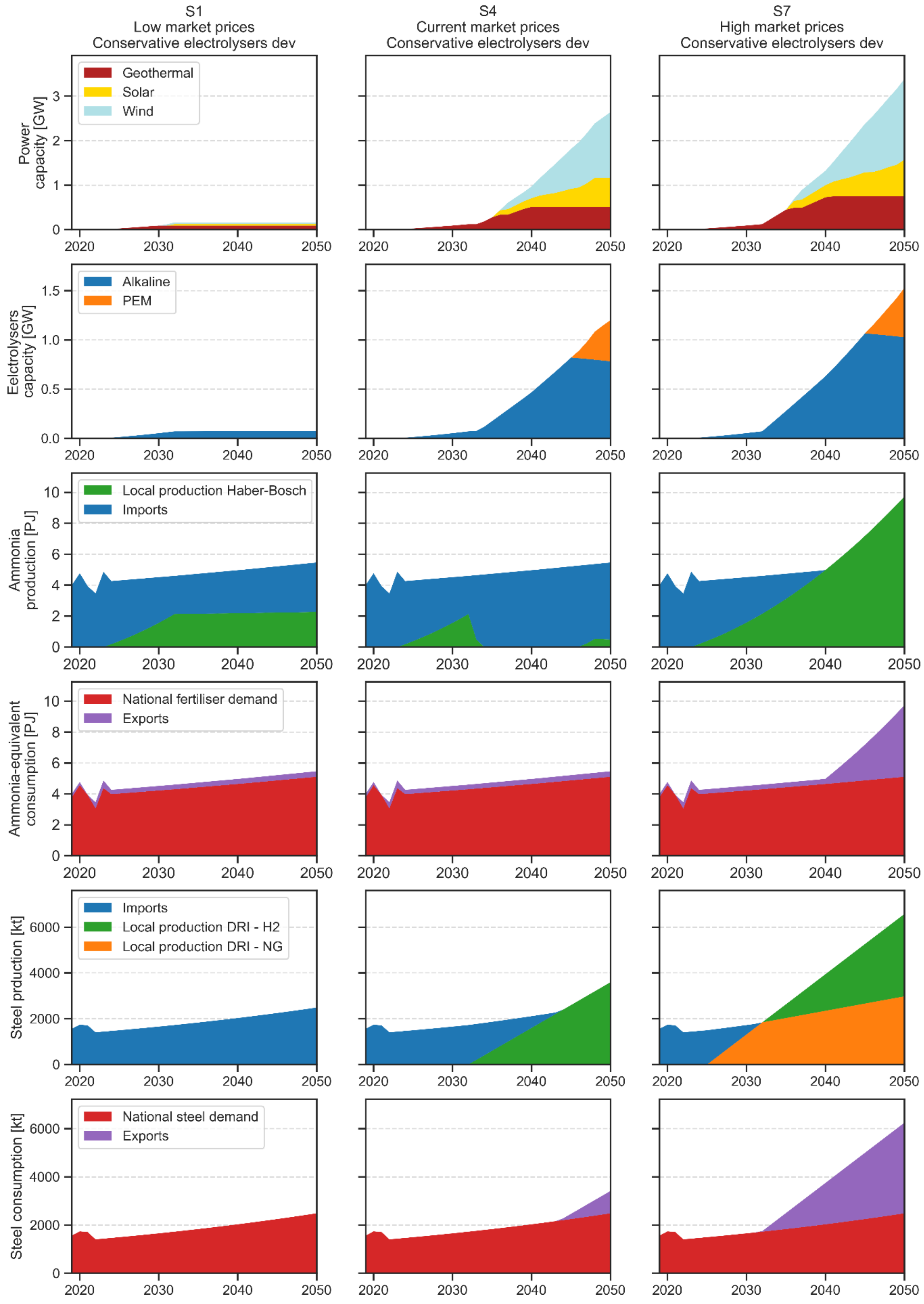

Figure S1 Results of runs based on low market prices for import/export of ammonia and steel (runs 1 to 3). Column 1 shows results for conservative hypothesis on the improvement of water electrolysis technologies, column 2 reference and column 3 optimistic.

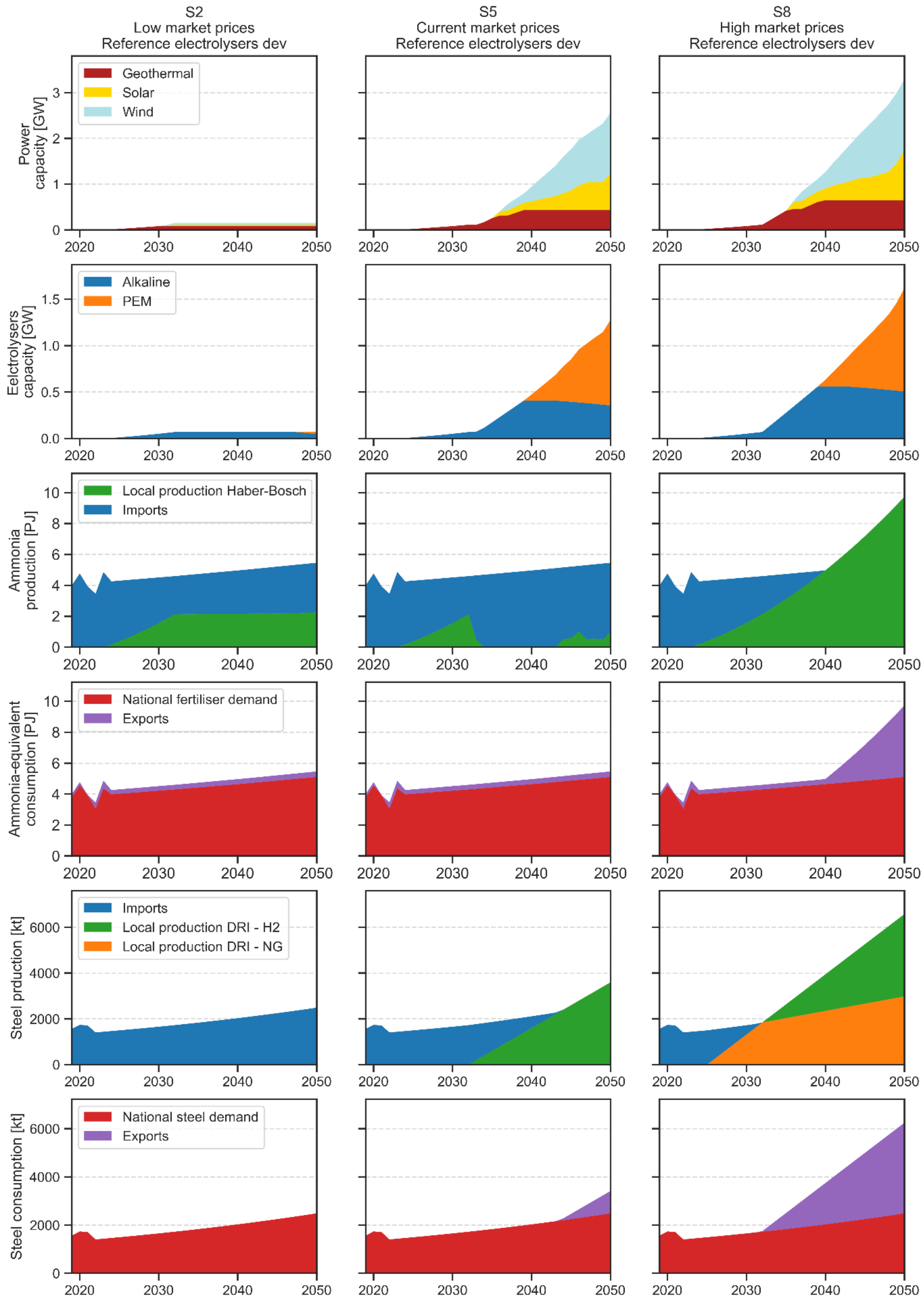

Figure S2 Results of runs based on mid market prices for import/export of ammonia and steel (runs 4 to 6). Column 1 shows results for conservative hypothesis on the improvement of water electrolysis technologies, column 2 reference and column 3 optimistic.

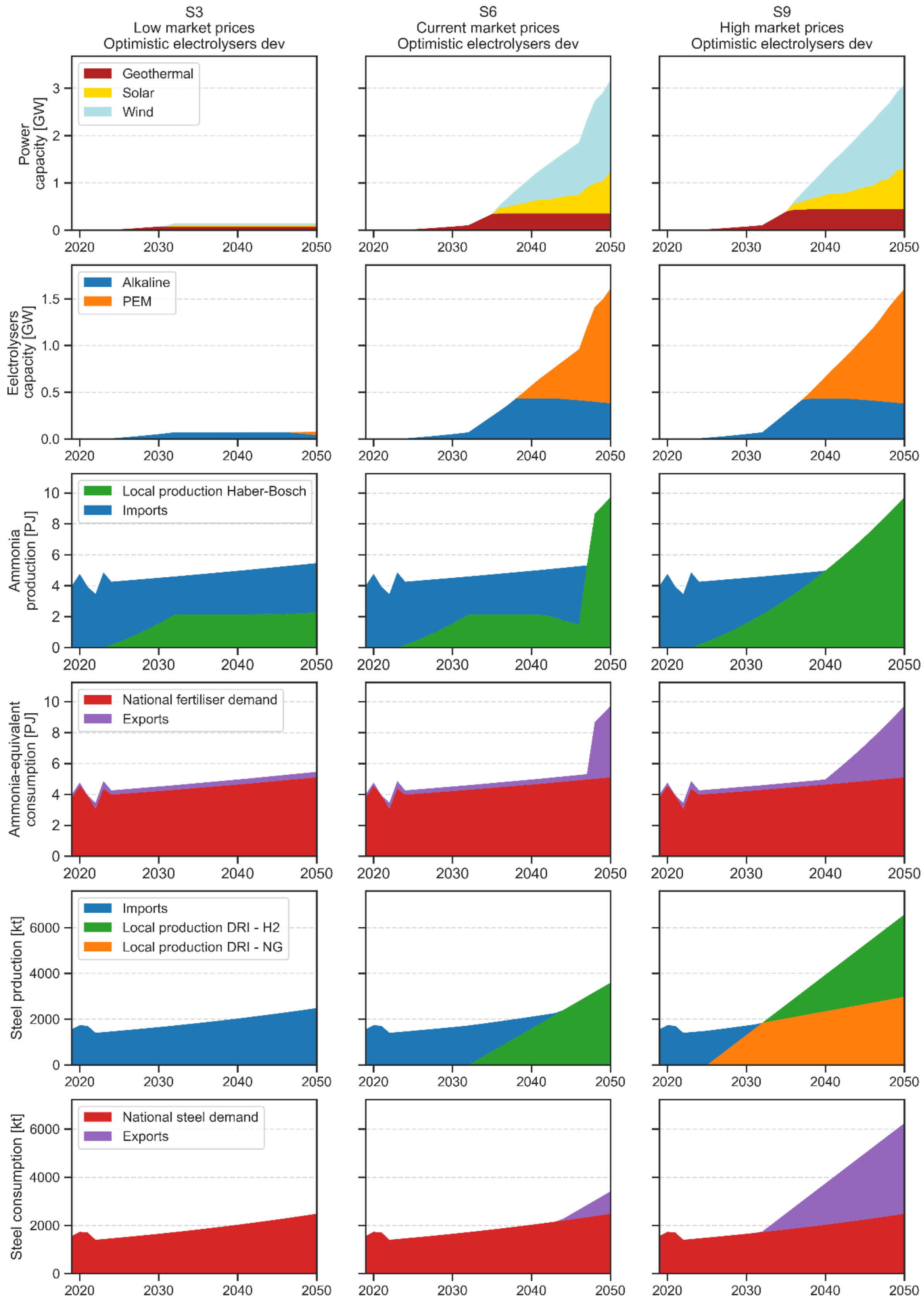

Figure S3 Results of runs based on high market prices for import/export of ammonia and steel (runs 7 to 9). Column 1 shows results for conservative hypothesis on the improvement of water electrolysis technologies, column 2 reference and column 3 optimistic.

Figures – Modelling assumptions

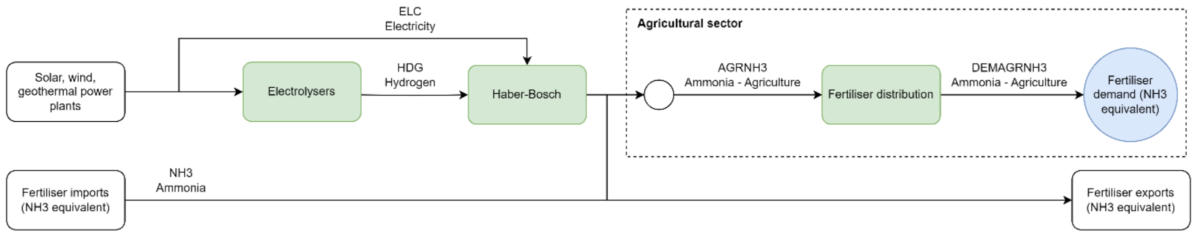

Figure 4 Reference energy system of the fertilizer subsector. Fertilizer demand, exports, and imports are represented through quantitites of ammonia containing the equivalent amount of nitrogen contained in the original mix of nitrogen-based fertilizers. Dedicated power from renewable energy sources is used to run the water electrolysis plant and the Haber-Bosch process, for the conversion of green hydrogen to ammonia.

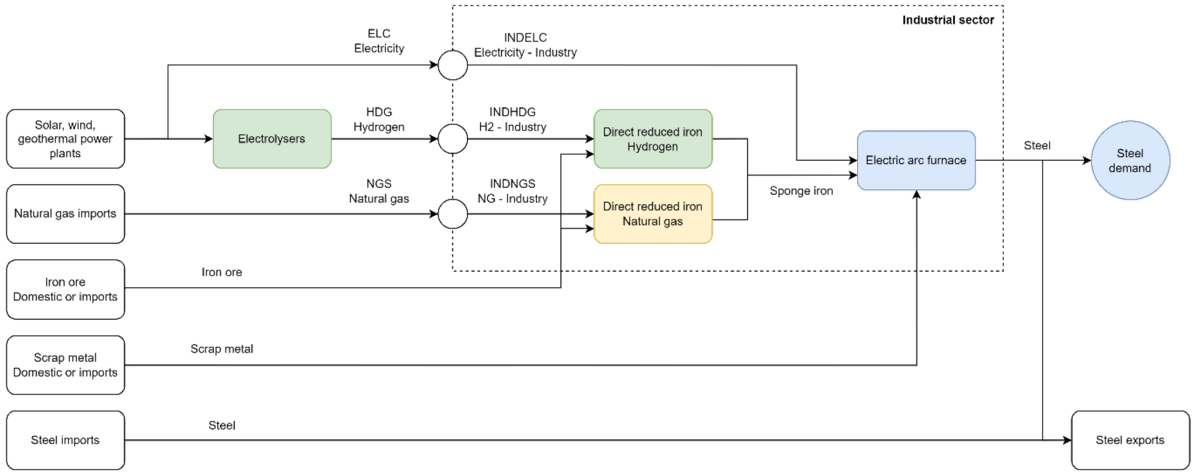

Figure 5 Reference energy system of the steel subsector. The electricity for green hydrogen production through water electrolysis and for the electric arc furnace (EAF) is provided by dedicated renewable energy source plants. Sponge iron produced through direct reduced iron based on hydrogen or natural gas is fed to the EAF for the production of steel that can be used as an alternative to imported steel to cover local demand or to be exported to the Eastern African market.

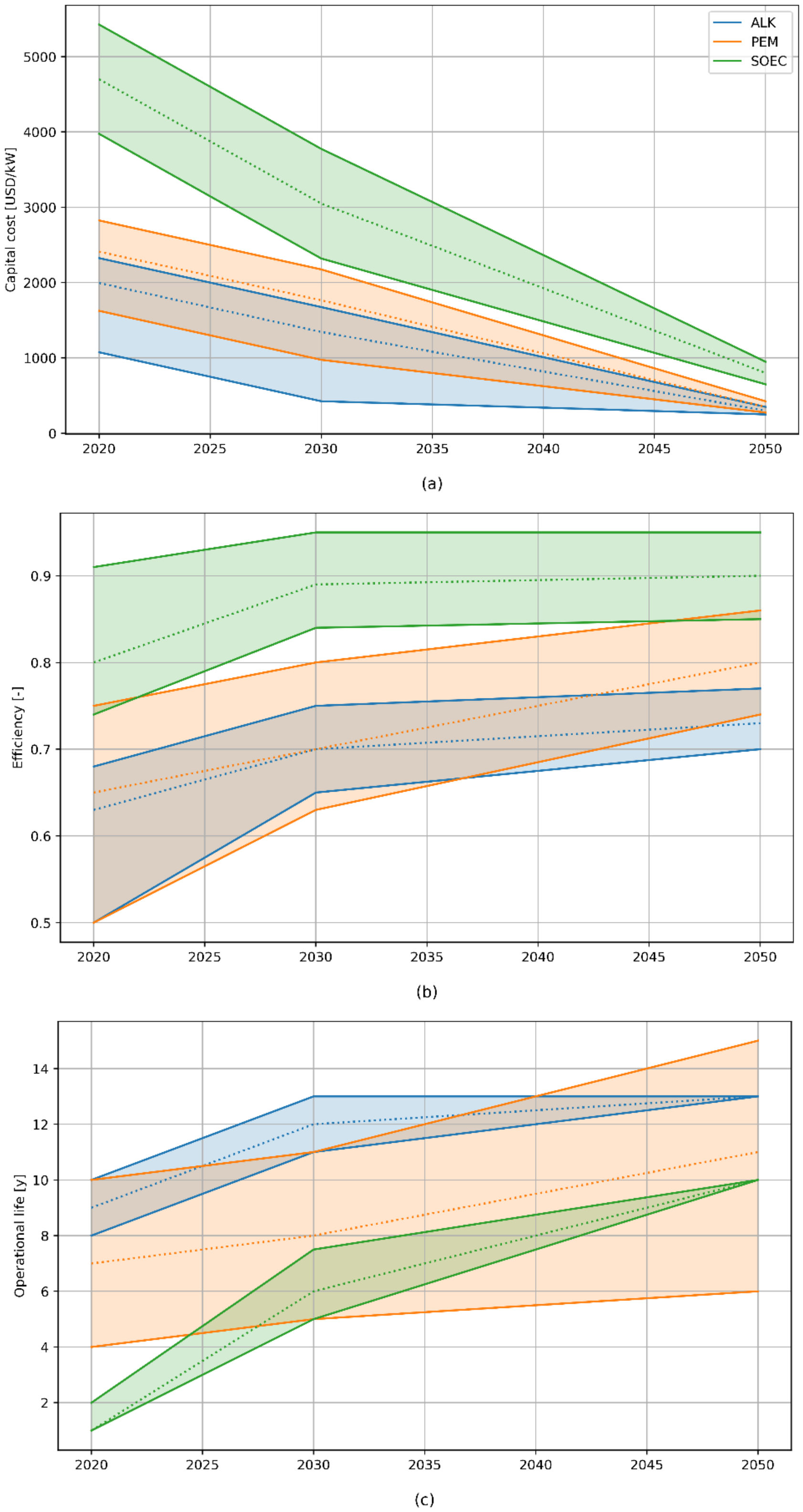

Figure S6 Projected cost reductions (a), improvements in efficiency (b) and improvements in operational stack lifetime for the alkaline (ALK), proton exchange membrane (PEM) and solid oxide electrolysis cells (SOEC) electrolysers. Based on the literature review summarised in Tables S2 to S4 and on the subsequent values listed in Tables S5 to S7.

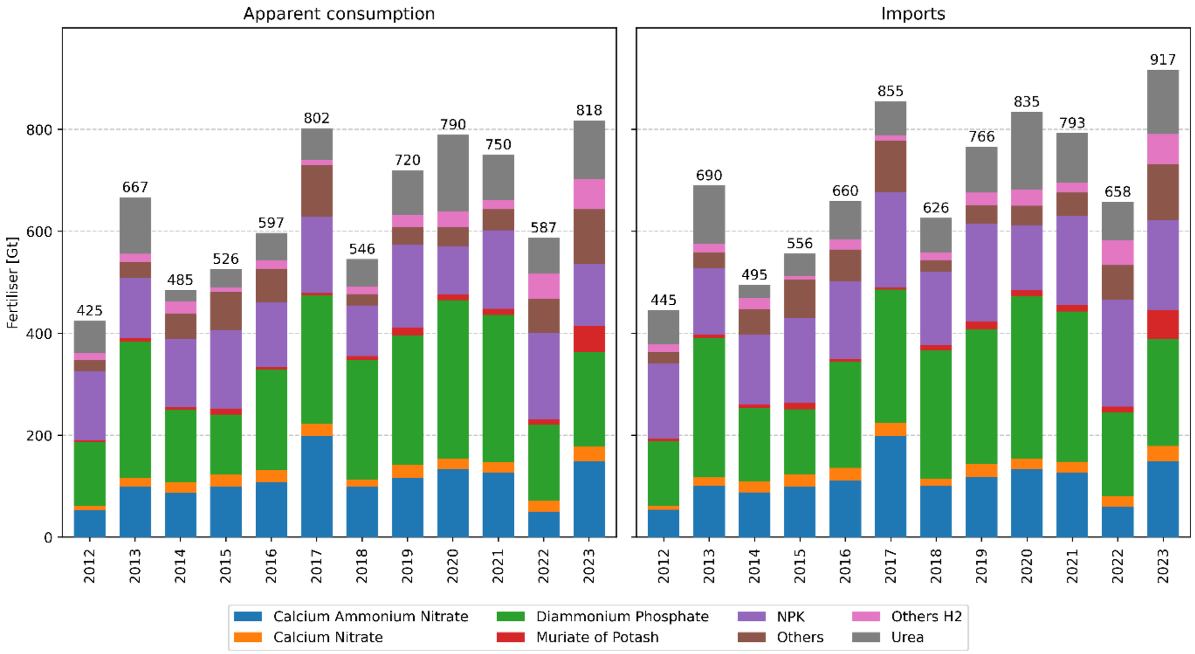

Figure S7 Annual fertilisers apparent consumption and imports in Kenya for the years 2012 to 2023.

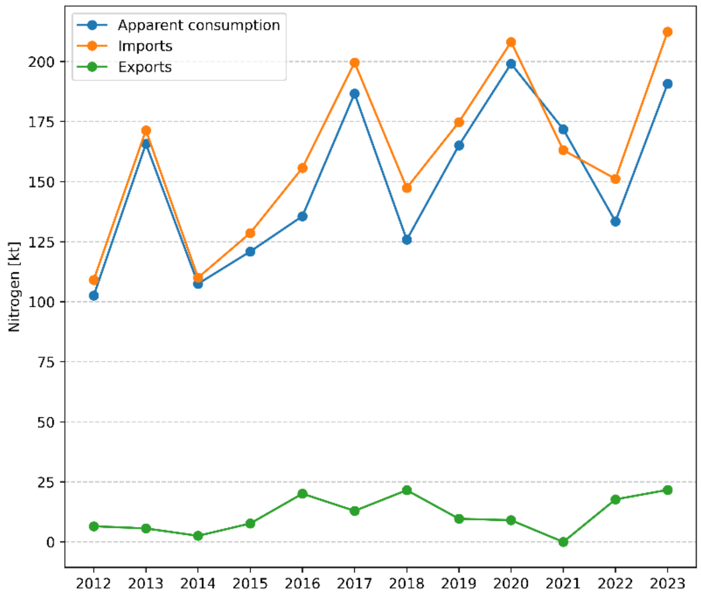

FigureS8 Annual nitrogen consumption, imports and exports in Kenya for the years 2012 to 2023.

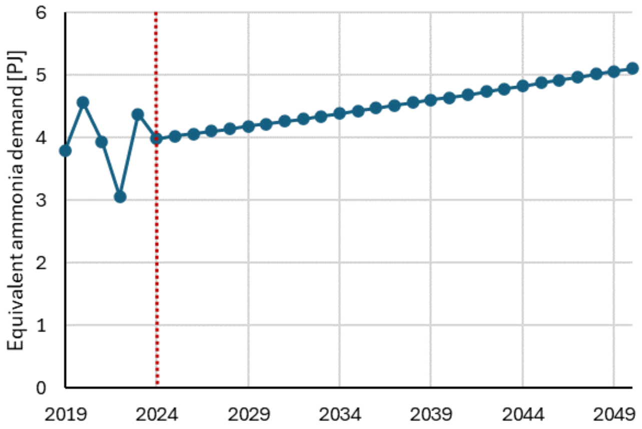

Figure S9 Demand projections for equivalent ammonia demand for nitrogen-based fertilisers in Kenya. The red dotted line separates historical data from the projected figures.

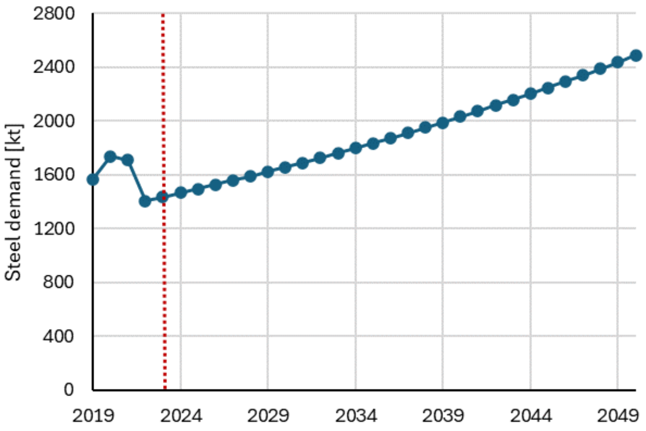

Figure S10 Demand projections for steel in Kenya. The red dotted line separates historical data from the projected figures.

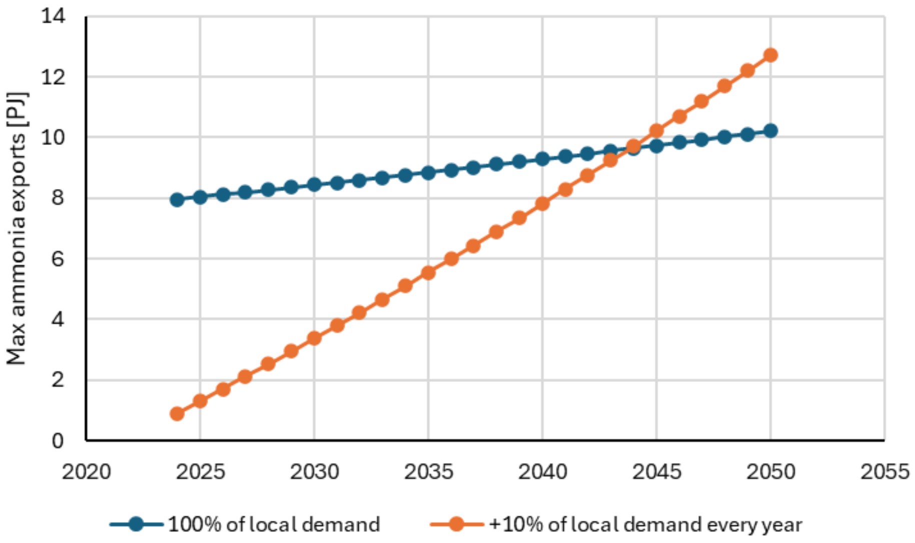

Figure S11 Constraint on maximum ammonia exports in the model. The actual constraint in the model corresponds to whichever constraint is more stringent between the two shown in the figure. The blue line corresponds to exports being equal to local annual demand, while the orange line corresponds to an increase of 10% of local demand every demand, starting from current exports levels.

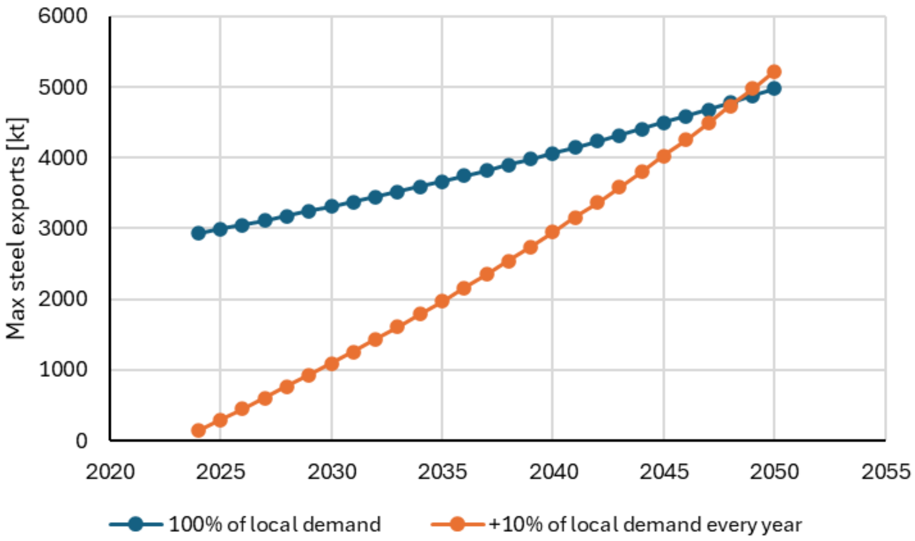

Figure S12 Constraint on maximum steel exports in the model. The actual constraint in the model corresponds to whichever constraint is more stringent between the two shown in the figure. The blue line corresponds to exports being equal to local annual demand, while the orange line corresponds to an increase of 10% of local demand every demand, starting from current exports levels.

Supplemental references

[S1] IEA, Global Hydrogen Review 2021: Assumptions Annex, 2021. https://iea.blob.core.windows.net/assets/2ceb17b8-474f-4154-aab5-4d898f735c17/IEAGHRassumptions_final.pdf.

[S2] IEA, Global Hydrogen Review 2023: Assumptions Annex, Paris, 2023. https://iea.blob.core.windows.net/assets/101dd112-b72b-4a74-82f1-de7fea6ae48e/GlobalHydrogenReview2023_AssumptionsAnnex.pdf.

[S3] IEA, Global Hydrogen Review 2024: Assumptions Annex, Paris, 2024.

[S4] Danish Energy Agency, Technology Data – Renewable fuels (Version 12 - Latest update April 2024), Copenhagen, 2024.

[S5] H. Blanco, W. Nijs, J. Ruf, A. Faaij, Potential for hydrogen and Power-to-Liquid in a low-carbon EU energy system using cost optimization, Appl Energy 232 (2018) 617–639. https://doi.org/10.1016/j.apenergy.2018.09.216.

[S6] ENTSO-E, Frontier Economics Ltd, Potential of P2H2 technologies to provide system services, Brussels, 2022. https://www.entsoe.eu/2022/06/28/entso-e-publishes-a-study-on-flexibility-from-power-to-hydrogen-p2h2/.

[S7] IRENA, Green Hydrogen Cost Reduction: Scaling up Electrolysers to Meet the 1.5^{0}C Climate Goal, Abu Dhabi, 2020. https://www.irena.org/publications/2020/Dec/Green-hydrogen-cost-reduction.

[S8] Danish Energy Agency, Technology Catalogues - Technology Data for Renewable Fuels - Version 7, Copenhagen, 2021. https://ens.dk/en/our-services/technology-catalogues/technology-data-renewable-fuels.

[S9] F.A. Plazas-Niño, R. Yeganyan, C. Cannone, M. Howells, B. Borba, J. Quirós-Tortós, Assessing the role of low-emission hydrogen: A techno-economic database for hydrogen pathways modelling, Data Brief 52 (2024) 109822. https://doi.org/10.1016/j.dib.2023.109822.

[S10] IEA, Ammonia Technology Roadmap – Towards more sustainable nitrogen fertiliser production, Paris, 2021. https://www.iea.org/reports/ammonia-technology-roadmap.

[S11] World Steel Association, Steel Statistical Yearbook - 2020 concise version, Brussels, 2020. https://worldsteel.org/wp-content/uploads/Steel-Statistical-Yearbook-2020-concise-version.pdf.

[S12] KNBS, Quarterly Balance of Payments 2019-2022, Nairobi, 2023. https://www.knbs.or.ke/button/quarterly-bop/.

[S13] F. Rosner, D. Papadias, K. Brooks, K. Yoro, R. Ahluwalia, T. Autrey, H. Breunig, Green steel: design and cost analysis of hydrogen-based direct iron reduction, Energy Environ Sci 16 (2023) 4121–4134. https://doi.org/10.1039/D3EE01077E.

[S14] Business Analytiq, Ammonia price index, Business Analytiq (2024). https://businessanalytiq.com/procurementanalytics/index/ammonia-price-index/.

[S15] Trading Economics, HRC Steel - Price - Chart - Historical Data, (2024). https://tradingeconomics.com/commodity/hrc-steel.
